# Machine learning approach identified clusters for patients with low cardiac output syndrome and outcomes after cardiac surgery

**DOI:** 10.3389/fcvm.2022.962992

**Published:** 2022-08-18

**Authors:** Xu Zhao, Bowen Gu, Qiuying Li, Jiaxin Li, Weiwei Zeng, Yagang Li, Yanping Guan, Min Huang, Liming Lei, Guoping Zhong

**Affiliations:** ^1^Department of Pharmaceutical Sciences, Institute of Clinical Pharmacology, Sun Yat-sen University, Guangzhou, China; ^2^Laboratory of South China Structural Heart Disease, Department of Intensive Care Unit of Cardiovascular Suregery, Guangdong Cardiovascular Institute, Guangdong Provincial People's Hospital, Guangzhou, China; ^3^Department of Pharmacy, The Second People's Hospital of Longgang District, Shenzhen, China

**Keywords:** artificial intelligence, machine learning, consensus clustering, low cardiac output syndrome, intensive care unit, individualized medicine

## Abstract

**Background:**

Low cardiac output syndrome (LCOS) is the most serious physiological abnormality with high mortality for patients after cardiac surgery. This study aimed to explore the multidimensional data of clinical features and outcomes to provide individualized care for patients with LCOS.

**Methods:**

The electronic medical information of the intensive care units (ICUs) was extracted from a tertiary hospital in South China. We included patients who were diagnosed with LCOS in the ICU database. We used the consensus clustering approach based on patient characteristics, laboratory data, and vital signs to identify LCOS subgroups. The consensus clustering method involves subsampling from a set of items, such as microarrays, and determines to cluster of specified cluster counts (k). The primary clinical outcome was in-hospital mortality and was compared between the clusters.

**Results:**

A total of 1,205 patients were included and divided into three clusters. Cluster 1 (*n* = 443) was defined as the low-risk group [in-hospital mortality =10.1%, odds ratio (OR) = 1]. Cluster 2 (*n* = 396) was defined as the medium-risk group [in-hospital mortality =25.0%, OR = 2.96 (95% CI = 1.97–4.46)]. Cluster 3 (*n* = 366) was defined as the high-risk group [in-hospital mortality =39.2%, OR = 5.75 (95% CI = 3.9–8.5)].

**Conclusion:**

Patients with LCOS after cardiac surgery could be divided into three clusters and had different outcomes.

## Introduction

Low cardiac output syndrome (LCOS) is a group of clinical syndromes characterized by decreased cardiac output and insufficient perfusion of peripheral organs, as seen in patients who have various disease processes, including shock, and those who have undergone cardiac surgery (1, 2). The diagnosis of LCOS was made if patients met more than two of the following diagnostic criteria: cardiac index < 2 L/min/m^2^; systolic blood pressure < 90 mm Hg or systolic blood pressure decreased by more than 20% compared with preoperative blood pressure; the difference between the central temperature and the peripheral temperature > 5 °C, and the limbs were cold; and urine volume < 0.5 ml/kg/h for more than 2 h. Besides, the mortality rates of patients with LCOS after cardiac surgery can exceed 20%. LCOS could result in prolonged hospitalization, increased complications, higher mortality rates, and higher medical expenses, and bring a heavy burden to patients and medical resources (3).

Several models for the outcomes of patients with LCOS had been established, such as the European System for Cardiac Operative Risk Evaluation (EuroSCORE), which predicts periodic cardiovascular alterations (4, 5). In addition, studies have shown that patients with LCOS after cardiac surgery may have potential patient types and different clinical outcomes. However, there was still a lack of research on the potential subgroups of patients with LCOS after cardiac surgery.

With the development of artificial intelligence, machine learning algorithm has been more and more widely used in personalized medicine (6–10). Supervised learning in machine learning is often used in the development and prediction of diseases, while unsupervised learning is used in the exploration of potential subgroups of diseases and the analysis of risk factors. A consensus clustering algorithm is a method that provides quantitative evidence for determining the number and membership of possible clusters within a dataset, such as a microarray gene expression. The consensus clustering method involves subsampling from a set of items, such as microarrays, and determines to cluster of specified cluster counts (k). Then, pairwise consensus values, the proportion that two things occupied the same cluster out of the number of times they occurred in the same subsample, are calculated and stored in a symmetrical consensus matrix for each *k* (11–13). In data analysis, the method of consensus clustering was used to generate stable results in a group of partitions provided by the random method. The consensus clustering method could improve the stability and accuracy of the partition results and provide more stable clustering results than the traditional methods. In the intensive care units (ICUs), this method could help doctors to find out different subtypes of patients and distinguish potential subtypes of diseases (14–17). More information about consensus clustering is given in Supplementary material 1.

This study aims to find the possible subtypes of patients with LCOS in the ICU database by the consensus clustering method and analyze the clinical outcomes of different clusters based on machine learning.

## Study design and enrollment

**Figure 1:**
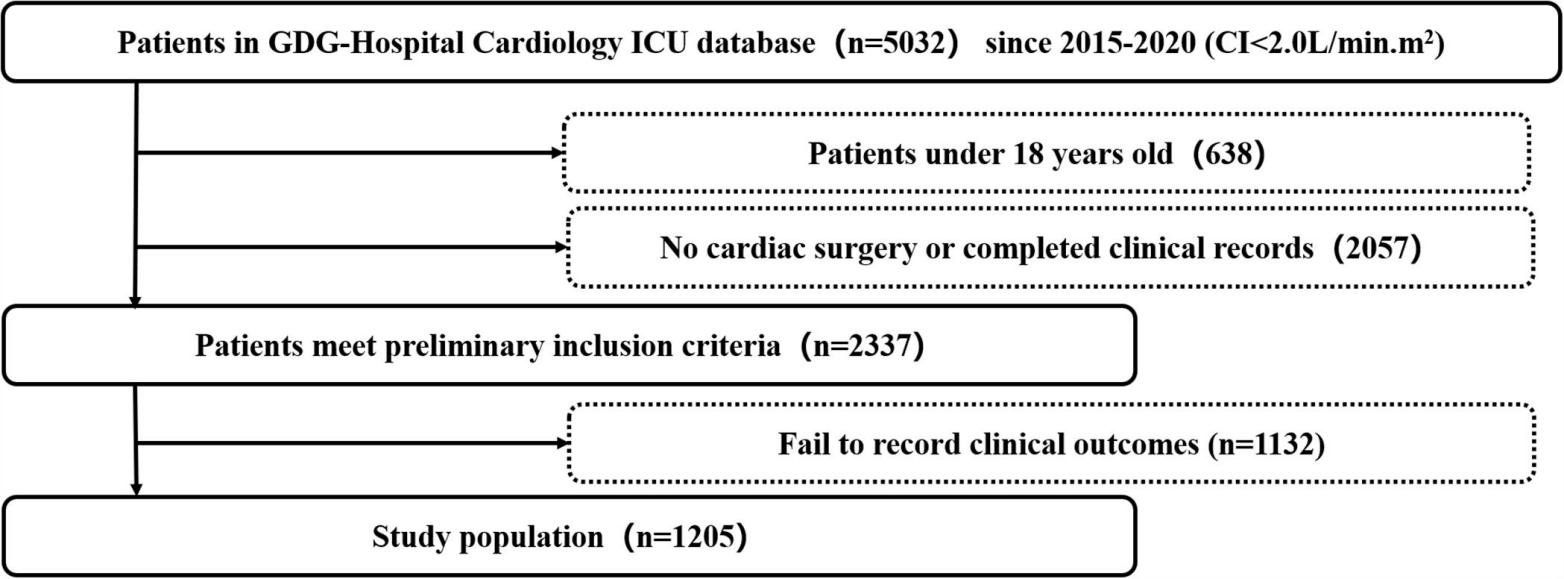
Flowchart of patients' selection.GDG hospital, Guangdong Provincial People Hospital; ICU, intensive care unit.

Data on clinical characteristics and outcomes were collected in a computerized database of 1,205 patients from the Cardiovascular Surgery Intensive Care Unit (CSICU) at Guangdong Provincial People's Hospital. The datasets were collected from 1 January 2016 to 1 October 2020. The clinical data were extracted from the ICU database for the study if they met the following inclusion criteria: (I) The patients were aged over 18 years old; (II) The patients had complete clinical outcomes; (III) The patients had undergone cardiac surgery; (IV) The patients were diagnosed with low cardiac output syndrome; (V) The clinical records of patients were filled over 90%; and (VI) The patients have undergone cardiac surgeries involving coronary artery bypass surgeries, and/or valve surgeries, aortic or major vascular surgeries with or without the use of extracorporeal circulation. The final clinical endpoint of interest is in-hospital mortality. The patient selection process is shown in Figure 1.

## Data extraction

We extracted a set of 19 variables from the datasets, including vital signs, laboratory test results, and patient's demographic information such as age, gender, weight, and body mass index (BMI). We chose the first recorded vital signs and laboratory values within 48 h after the patients were admitted to the CSICU. The k-nearest neighbors algorithm (KNN) was used to fill the missing value for patients who lacked <10% clinical data (18–20). Our data include continuous and subtype variables. We used one hot coder method with an unstable feature vector, which could deal with the complex features and expand the vectors, to make the models more stable. The processed scalars were standardized to cluster the multidimensional features in the same dimension, and to reduce the excessive effect of some features.

An unsupervised machine learning approach was used to identify the potential subtypes of patients with LCOS. The consensus clustering method was defined by a prespecified subsampling parameter of 80% with 400 iterations and the potential clusters (k) to range from 2 to 10 to avoid too many clusters that are not clinically useful (21–24). The algorithm was chosen by partitioning around medoids (PAM) to avoid outliers (20). We chose the optimal *k* by consensus cluster plots, delta area plots, heat maps, and cumulative distribution function maps. The consensus score within the cluster, ranging from 0 to 1, is defined as the average consensus value of all the individual pairs belonging to the same cluster. The closer the value is to one, the better the stability of the cluster. The clinical outcome of patients was in-hospital mortality and we analyzed and compared the main outcomes of patients after clustering.

## Statistical methods

After using the clustering method to determine the potential types of samples, we compared the differences in clinical results. We used the ANOVA test and chi-squared test to verify the difference between continuous variables and categorical variables. We took the average value of each cluster as the representative of the whole cluster and compared its differences. Then, we tested the hypothesis of in-hospital mortality among different clusters and calculated the relative risk ratio with the lowest risk cluster as the control. All the statistical analyses were performed using SPSS (version 25.0) and R (version 4.2.0) (RStudio, 2 February 2022) under Windows system (professional version 10), with packages of BiocManager (version 1.30.17) and ConsensusClusterPlus (version 1.60.0) (25).

## Results

### Patients' characteristics

A total of 1,205 patients from the CSICUs at Guangdong Provincial People's Hospital were included in this study. There were 454 women (30.2%) and 751 men (69.8%) with an average age of 55.5 ± 12.7 years. The overall patients' characteristics are shown in Table 1.

**Table 1:** Clinical characteristics at ICU admission of patients with low cardiac output syndrome.

**Parameters**	**Overall**	**Cluster 1**	**Cluster 2**	**Cluster 3**
	**(*****n** **=*** **1205)**	**(*****n** **=*** **366)**	**(*****n** **=*** **396)**	**(*****n** **=*** **443)**
Age	55.5 ± 12.7	57.9 ± 12.1	52.6 ± 13.8**	59.8 ± 11.7^*, *##*^
Male sex	751 (70)	46 (13)	303 (77) *	402 (91) ^*, #^
Weight (kg)	60.2 ± 10.3	55.1 ± 9.2	62.3 ± 9.5**	63.4 ± 10.0^**, #^
BMI	21.2 ± 5.6	19.6 ± 3.4	22.4 ± 10.6*	22.6 ± 8.4^*, #^
**Vital signs**				
—Heart rate(per min)	94.1 ± 15.5	86.7 ± 12.6	102.2 ± 15**	93.0 ± 14.9^**, *##*^
—Systolic blood pressure(mmHg)	95.5 ± 18.4	98.8 ± 20.2	91.4 ± 17.5**	90.9 ± 14.9^**, #^
—Diastolic blood pressure(mmHg)	67.1 ± 12.0	70.4 ± 11.7	64.1 ± 11.7*	66.9 ± 11.7^*, #^
—Temperature (F)	38.2 ± 0.7	38 ± 0.7	37.8 ± 0.7*	39.0 ± 0.7^*, #^
—Oxygen saturation(%)	95.9 ± 3.9	96.9 ± 3.9	97.5 ± 3.4*	96.4 ± 4.2^*, #^
**Laboratory examinations**				
—White blood cell( ×10^9^)	15.3 ± 5.1	13.6 ± 4.5	17.0 ± 5.0**	15.0 ± 4.8^*, #^
—Platelet( ×10^9^)	96.5 ± 49.6	75.3 ± 36.3	111.7 ± 5.6**	100.3 ± 49.6^**, *##*^
—International normalized ratio	2.25 ± 1.87	1.97 ± 0.7	2.53 ± 2.8**	2.23 ± 1.4^*, #^
—Prothrombin time(per s)	23.8 ± 8.9	20.8 ± 7.1	24.9 ± 9.6**	25.3 ± 9.1^**, #^
—Albumin(g/L)	41.8 ± 29.7	46.1 ± 29.7	40.4 ± 21.2**	39.2 ± 19.1^**, #^
—Total bilirubin(g/L)	50.3 ± 54.5	46.3 ± 42.8	51.0 ± 43.4**	53.1 ± 56.8^**, *##*^
—Urea (mmol/L)	19.7 ± 11.4	15.9 ± 8.6	21.1 ± 11.9**	21.5 ± 12.6^**, #^
—Creatinine (μmoI/L)	196.5 ± 146.3	153.1 ± 105.6	217.5 ± 160.7**	213.5 ± 154.9^**, #^
—ALT(U/L)	136.9 ± 45.8	89.6 ± 25.9	168.5 ± 47.1**	228.7 ± 56.4^**, *##*^
—AST(U/L)	243.7 ± 64.9	141.8 ± 40.2	254.7 ± 69.0**	317.2 ± 75.7^**, *##*^

Continuous variables were reported as mean ± standard deviation. BMI, body mass index. (1) Post-hoc p-values for pairwise comparisons between the clusters are provided within panels. ^*^P < 0.01, ^**^P < 0.001 vs. cluster 1. ^#^P < 0.01, ^##^P < 0.001 vs. cluster 2.

### Three main clusters were identified in patients with low cardiac output syndrome

**Figure 2:**
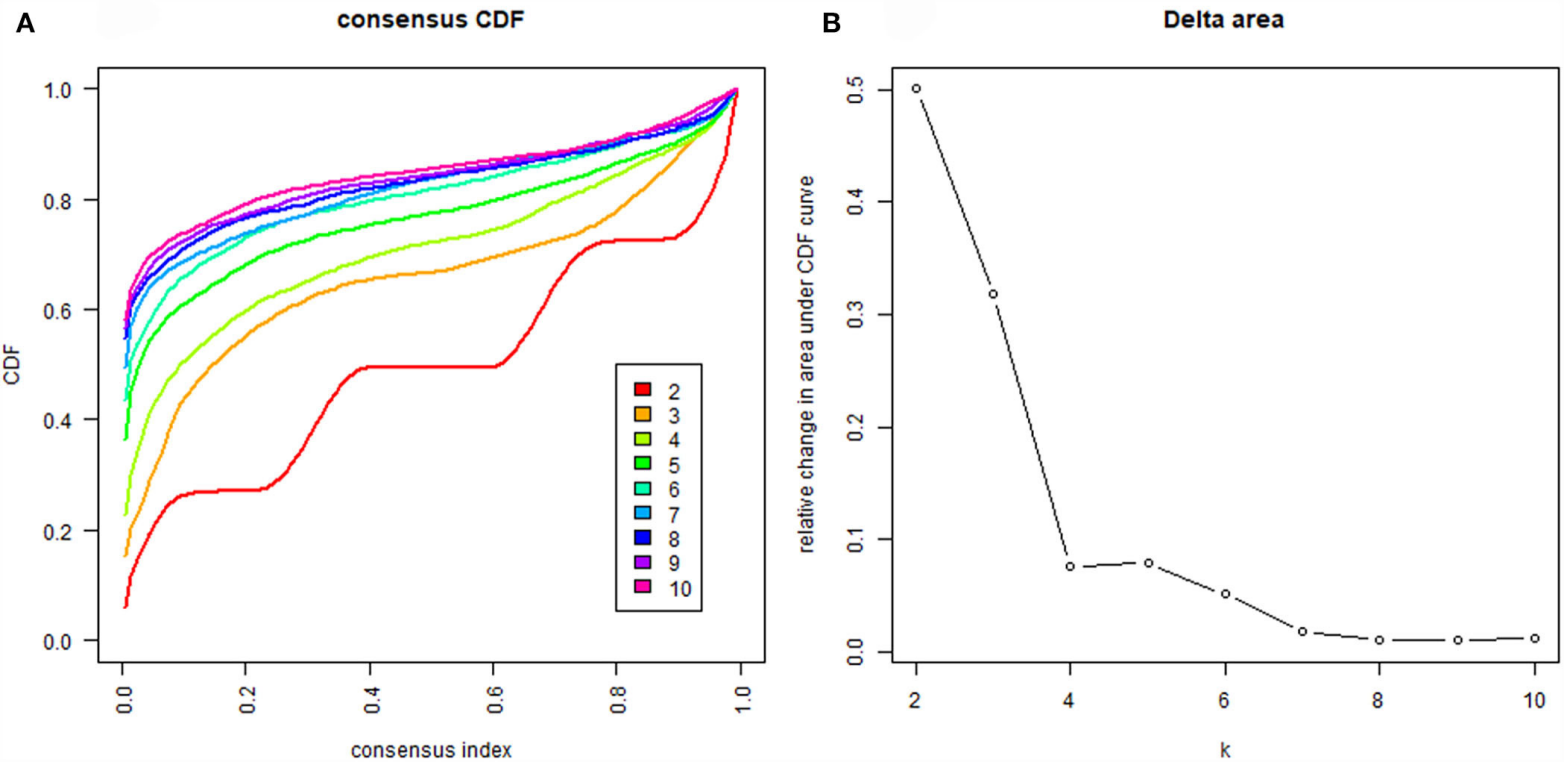
**(A)** CDF shows the cumulative distribution function when *k* takes different values for patients with low cardiac output syndrome; and **(B)** Delta area plot shows the relative change of the area under the CDF curve compared with *k* and k-1.

**Figure 3:**
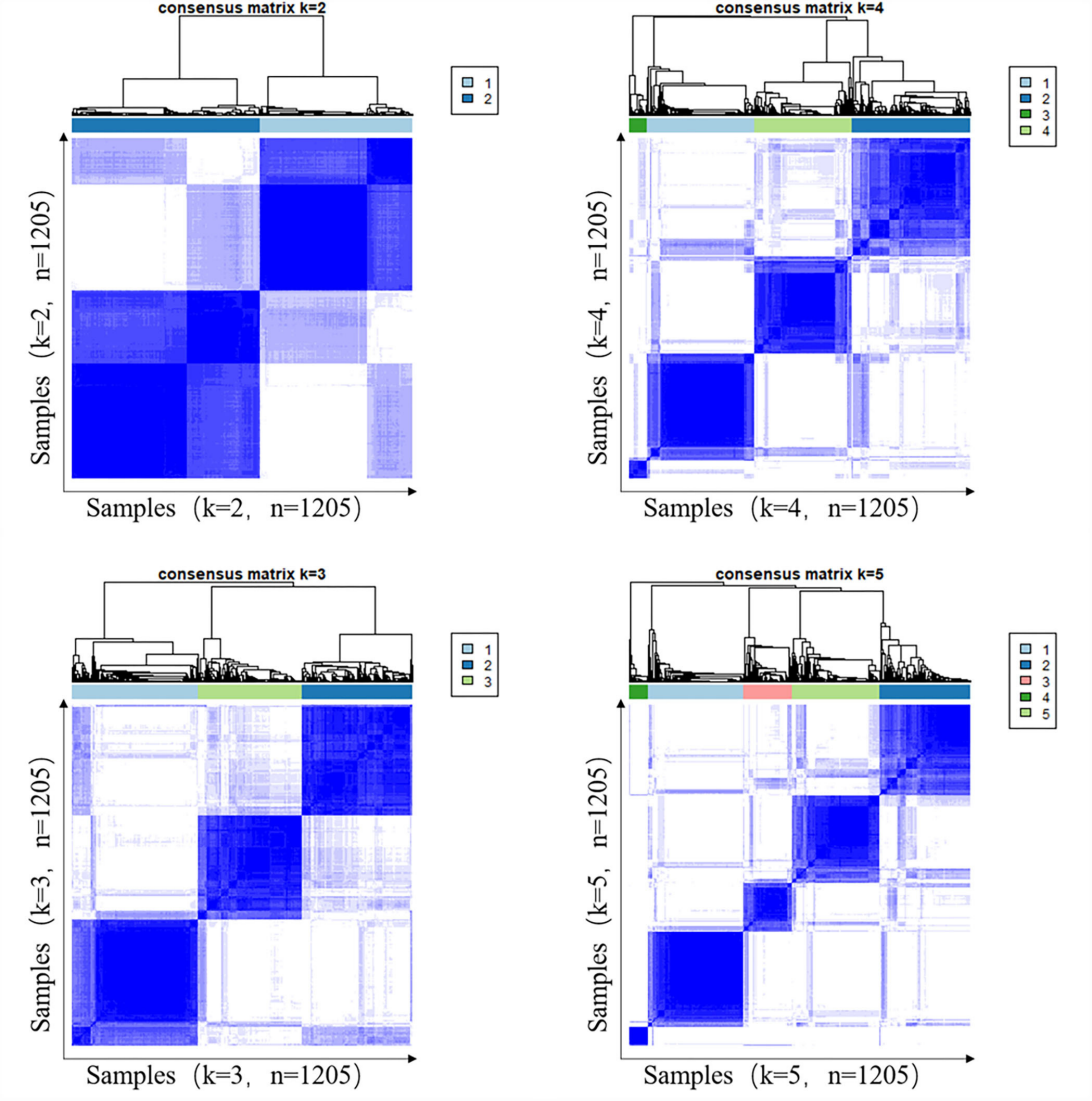
The consensus matrix heat map depicts the consensus value of each cluster in a white to blue color scale.

**Figure 4:**
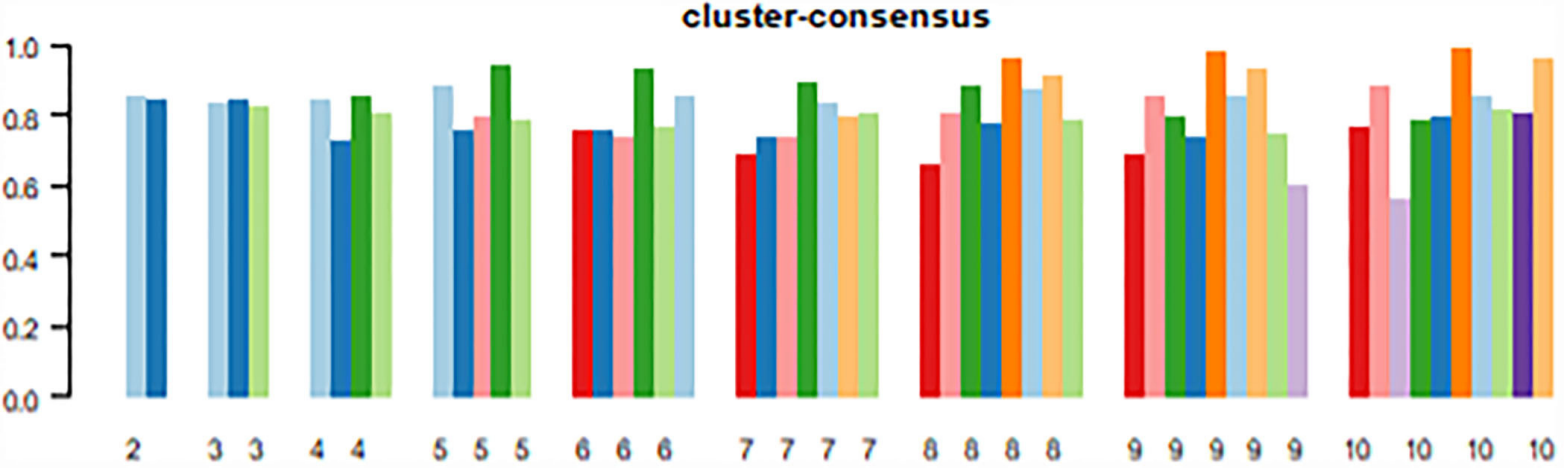
This cluster consensus plot shows the cluster consumption value of each category under different *k* values.

After 400 repeated iterations of patient data, the cluster results of patients from *k* = 2 to *k* = 10 were obtained. The cumulative distribution function (CDF) plot (Figure 2A) shows the cumulative distribution function when *k* takes different values. The smoother the overall curve changes, the more stable and accurate the classification effect would be. The delta area plot (Figure 2B) shows the relative change of the area under the CDF curve compared with values *k* and k-1. The largest change occurred when *k* = 2 and *k* = 3, which is meaningful. As shown in heat maps (Figure 3), the consensus clustering method found cluster 3 and cluster 4 with relatively clear boundaries, which indicates good stability over repeated iterations. The smaller the area difference of the blue area, the clearer the white of the edge part, and the more stable and accurate the overall classification effect. The average cluster consensus plot (Figure 4) points got relatively high when *k* = 2 or *k* = 3. Finally, we chose the *k* value of three to represent the data pattern of patients with LCOS based on the figures and clinical meaning. We provide detailed information for the figures in Supplementary material 1.

After the clusters were classified, there were 443 patients in cluster 1, 396 patients in cluster 2, and 366 patients in cluster 3. The patients' characteristics were significantly different among groups (shown in Table 1).

The proportion of men in cluster 1 was 13% (46/366) and lower than cluster 2 (*p* < *0.001*) and cluster 3 (*p* < *0.01*). The average body weight (55.1 ± 9.2 kg) and BMI (19.6 ± 3.4) of cluster 1 were lower than cluster 2 (*p* < *0.001*) and cluster 3 (*p* < *0.001*). For vital signs, cluster 1 had lower heart rate (86.7 ± 12.6, *p* < *0.001 vs. cluster 2 and p* < *0.001 vs. cluster 3*), but higher systolic blood pressure (98.8 ± 20.2, *p* < *0.001 vs. cluster 2 and p* < *0.001 vs. cluster 3*) and diastolic blood pressure (70.4 ± 11.7, *p* < *0.01 vs. cluster 2 and p* < *0.01 vs. cluster 3*). As for laboratory data, cluster 1 patients had lower white blood cells (13.6 ± 4.5), platelets (75.3 ± 36.3), international normalized ratio (INR) value (1.97 ± 0.7), PT value (20.8 ± 7.1), albumin (46.1 ± 29.7), total bilirubin (46.3 ± 42.8), urea (15.9 ± 8.6), creatinine (153.1 ± 105.6), alanine aminotransferase (ALT) (89.6 ± 25.9), and aspartate aminotransferase (AST) (141.8 ± 40.2) (*p* < *0.001 vs. cluster 2 and p* < *0.001 vs. cluster 3*).

The proportion of men in cluster 2 (303/396, *p* < *0.01 vs. cluster 1 and p* < *0.01 vs. cluster 3*) was much higher than in others. The second cluster was characterized by lower heart rate (102.2 ± 15.0, *p* < *0.001 vs. cluster 1 and p* < *0.001 vs. cluster 3*) and diastolic blood pressure (91.4 ± 17.5, *p* < *0.01 vs. cluster 1 and p* < *0.01 vs. cluster 3*), but higher platelet (117.7 ± 5.6, *p* < *0.001 vs. cluster 1 and p* < *0.001 vs. cluster 3*), INR value (2.53 ± 2.8, *p* < *0.001 vs. cluster 1 and p* < *0.001 vs. cluster 3*), and albumin (40.4 ± 21.2, *p* < *0.001 vs. cluster 1 and p* < *0.001 vs. cluster 3*). On the whole level, the data of cluster 2 were in the middle state relative to cluster 1 and cluster 3.

The average age (59.8 ± 11.7, *p* < *0.01 vs. cluster 1 and p* < *0.001 vs. cluster 2*), body weight (63.4 ± 10.0, *p* < *0.001 vs. cluster 1 and p* < *0.01 vs. cluster 2*), and BMI (22.6 ± 8.4, *p* < *0.01 vs. cluster 1 and p* < *0.01 vs. cluster 2*) of patients in cluster 3 were higher. For vital signs, patients in cluster 3 were characterized by the highest temperature (39.0 ± 0.7, *p* < *0.001 vs. cluster 1 and p* < *0.001 vs. cluster 2*), but lower systolic blood pressure (90.9 ± 14.9, *p* < *0.001 vs. cluster 1 and p* < *0.01 vs. cluster 2*). Cluster 3 had higher PT levels (25.3 ± 9.1, *p* < *0.001*), total bilirubin level (53.1 ± 56.8), urea (21.5 ± 12.6), creatinine (213.5 ± 154.9), ALT (228.7 ± 56.4), and AST (317.2 ± 75.7) (*p* < *0.001 vs. cluster 1 and p* < *0.001 vs. cluster 2*).

The in-hospital mortality was 10.1, 25, and 39.2% in cluster 1, cluster 2, and cluster 3, respectively (Table 2). Cluster 3 had the highest in-hospital mortality compared to cluster 1 and cluster 2. Cluster 3 (OR 5.75, 95% CI 3.9–8.5) was significantly associated with higher in-hospital mortality compared to cluster 1 and cluster 2 (Table 2).

**Table 2:** Clinical outcomes according to clusters of patients with low cardiac output syndrome.

**Cluster**	**In-hospital mortality**	
	**%**	**OR (95% CI)**
Overall	25.7%	-
Cluster 1	10.1%	1 (ref)
Cluster 2	25%	2.96 (1.97–4.46)
Cluster 3	39.2%	5.75 (3.9–8.5)

OR, Odds Ratio.

## Discussion

Compared with other diseases, the definition of low cardiac output syndrome was more extensive, which included patient's cardiac index was lower than 2.0 L/min/m^2^, usage of intra-aortic balloon pump (IABP) after cardiac surgery, and the usage of inotropic medication (either dopamine, dobutamine, milrinone, or epinephrine) that affect cardiac contraction after cardiac surgery (2, 3). This study included patients based on a wide range of heart index lower than 2.0 L/min/m^2^ and excluded the interference of other confounding factors. For patients with low cardiac output syndrome, the pathophysiological mechanism had been clearly described. However, there was still a lack of discussion on potential subtypes or risk groups of patients with LCOS after cardiac surgery, so we used the consensus clustering method based on the unsupervised study in machine learning, which could be applied to a large number of patients with different characteristics in the dataset to identify the potential subgroups. Compared with the previous multifactors correlation analysis, the large number of repeated iteration processes from the consensus clustering method ensured that the results were more stable and reliable (1). In this study, we classified three potential clusters through the consensus clustering method. There were significant differences in clinical characteristics and outcomes of the three clusters. We finally defined the three clusters as the low-, medium-, and high-risk groups, respectively.

### Impact of patients' characteristic

The in-hospital mortality of patients in the high-risk group was close to 40%, which was not only higher than the low-risk and medium-risk groups, but also higher than patients with LCOS after cardiac surgery reported in the literature before. It had been reported that gender was an independent risk factor from 1990 to 1999 for patients with LCOS. However, gender was no longer an independent risk factor after 2000 and was replaced by malnutrition (2, 3). In this study, the proportion of men in the low-risk group was significantly lower than in the medium- and high-risk groups. On the other hand, the high-risk group has the highest proportion of men and in-hospital mortality. Our research suggested a possibility that gender may still be a potential risk factor and index for patients with LCOS after cardiac surgery.

Related articles reported that obesity was a risk factor for heart failure in patients after cardiac surgery (26). However, there was still a lack of evidence that higher body weight and body mass index were risk factors for patients with LCOS after cardiac surgery. On the other hand, the body mass index of the three clusters was all under the obesity warning line, which may suggest that body weight and body mass index were not noteworthy factors. Besides, the proportion of women in the low-risk group in this study was higher than in the medium-risk and high-risk groups, which might lead to the lower BMI of patients in the low-risk group.

According to previous articles, age older than 65 years old was considered a risk factor for patients with LCOS after cardiac surgery (2, 3). However, the average age of the high-risk group in this study was 60 years old and lowers than the previous dangerous line. Furthermore, the in-hospital mortality of the high-risk group was higher than the previous patients with age over 65 years old. Our study suggested that the dangerous line of ages for patients with LCOS after cardiac surgery should be downward adjusted. This research served as a proof of concept that age was still a risk factor for patients with LCOS after cardiac surgery, and the potential risk age of the patient population showed a downward trend.

### Impact on body temperature and systolic blood pressure

The average body temperature of patients in the high-risk group was significantly higher than the low-risk and medium-risk groups, which may be related to the increase in body temperature caused by postoperative infection. It had been reported that high body temperature may be related to the increased mortality of patients after cardiac surgery, but whether it was related to the death of patients with low cardiac output syndrome after cardiac surgery is still controversial (27). Our results suggested a possibility that body temperature was a noteworthy risk factor for patients with LCOS after cardiac surgery, especially for patients' body temperature over 39°C.

The systolic blood pressure of patients in the low-risk group with LCOS after cardiac surgery was significantly higher than in the medium-risk and high-risk groups. Values of systolic blood pressure were highly positively correlated with left ventricular ejection fraction (28). It was considered that patients with lower left ventricular ejection fraction were a high risk population, especially for patients with LCOS after cardiac surgery (1, 3–5). This study suggests that systolic blood pressure could be used as an alternative prognostic indicator when the left ventricular ejection fraction could not be effectively monitored.

### Impact of laboratory examinations

Considering that cardiovascular disease and cardiac surgery cause profound alterations in systemic metabolism and endocrine function, many trials have been conducted to determine the biochemical predictors of patients with LCOS after cardiac surgery. However, there was still a controversy about potential biochemical predictors of patients with LCOS. Our studies showed that the albumin content of the low-risk group was significantly higher than the medium-risk and high-risk groups. Related articles showed that patients with lower albumin level after cardiac surgery had worse clinical outcomes than others, especially for patients with albumin under 40 g/l (29). Considering that the low-risk group has the highest albumin levels and the high-risk group has the lowest albumin level, we believed that a higher albumin level was an indicator of good prognosis of patients with LCOS after cardiac surgery.

Aspartate aminotransferase and ALT proteins in the high-risk group were higher than in the medium-risk group and the low-risk group. Considering that ALT proteins were more specific than AST proteins and higher ALT proteins were highly positively correlated with mortality of patients with liver function damage after cardiac surgery, we suggested a possibility that a higher level of ALT proteins could be a risk factor for patients with LCOS after cardiac surgery.

Overall, we used an optimized clustering algorithm for unsupervised cluster analysis of the ICU patients with LCOS. The optimized algorithm could avoid unstable clustering results due to the existence of outliers (30, 31). We classified three possible clusters in patients with LCOS after cardiac surgery, and compared their clinical outcomes. The clinical outcomes of the three clusters were significantly different, suggesting the potential risk factors in patients with LCOS after cardiac surgery. Our study suggested that there might be potential subtypes in patients with LCOS after cardiac surgery. Compared with the traditional EuroSCORE method, we also proposed a new method to systematically evaluate patients through machine learning (27, 32).

### Study limitations

There were some limitations to our study. First, our study did not include physiological indexes such as pH values and we lacked some subjective clinical scores of patients in the study. Second, we did not include the clinical indicators before or after the ICU admission and the dynamic time series data of patients. Third, our study still needs more patients' records to establish a death prediction model for patients with LCOS based on classified clusters. Finally, all the cardiac surgeries with complete clinical data that were admitted to the intensive care unit were included in the study, and we did not consider looking for LCOS in patients with or without the use of extracorporeal circulation. We think that it can be studied in future clinical studies.

## Conclusion

In conclusion, we classified three possible clusters of patients with LCOS using an unsupervised machine learning algorithm in the dataset. We compared the clinical outcomes of these three clusters and found their differences. Our future study will further improve the machine learning method to explore the individualized treatment of patients with ICU with LCOS and establish a death prediction model for patients with LCOS based on the consensus clustering method.

## Data availability statement

The data analyzed in this study is subject to the following licenses/restrictions: joint approval of hospital and school is required to submit the datasets. Requests to access these datasets should be directed to XZ, 857957280@qq.com.

## Ethics statement

The study involving human participants was reviewed and approved by the Ethics Committee of the Guangdong Provincial People's Hospital, Guangzhou, China. The patients/participants provided their written informed consent to participate in this study.

## Author contributions

LL and GZ were responsible for funding acquisition, and manuscript review. MH was responsible for manuscript editing. YL and YG were responsible for project administration. QL and JL were responsible for supervision. XZ and BG were responsible for writing the original draft, formal analysis, software, investigation, and visualization. All authors contributed to the article and approved the submitted version.

## Funding

The authors appreciate the financial support provided by the National Natural Science Foundation of China (No. 82173776), the Natural Science Foundation of Guangdong Province (No. 2021A1515010574), the Key Laboratory Project of Guangdong Medical Products Administration (No. 2021ZD808), the Guangdong Provincial Key Laboratory of Construction Foundation (No. 2020B1212060034), and the National Key Research and Development Program (No. 2020ZX09201-021).

## Conflict of interest

The authors declare that this study was conducted in the absence of any commercial or financial relationships that could be construed as the potential conflicts of interest.

## Publisher's note

All claims expressed in this article are solely those of the authors and do not necessarily represent those of their affiliated organizations, or those of the publisher, the editors and the reviewers. Any product that may be evaluated in this article, or claim that may be made by its manufacturer, is not guaranteed or endorsed by the publisher.
