# Supplementary figures and images for: Machine learning approach identified clusters for patients with low cardiac output syndrome and outcomes after cardiac surgery
Source: Front Cardiovasc Med. 2022 Aug 18;9:962992. doi: 10.3389/fcvm.2022.962992 (PMC9434347; doi:10.3389/fcvm.2022.962992)

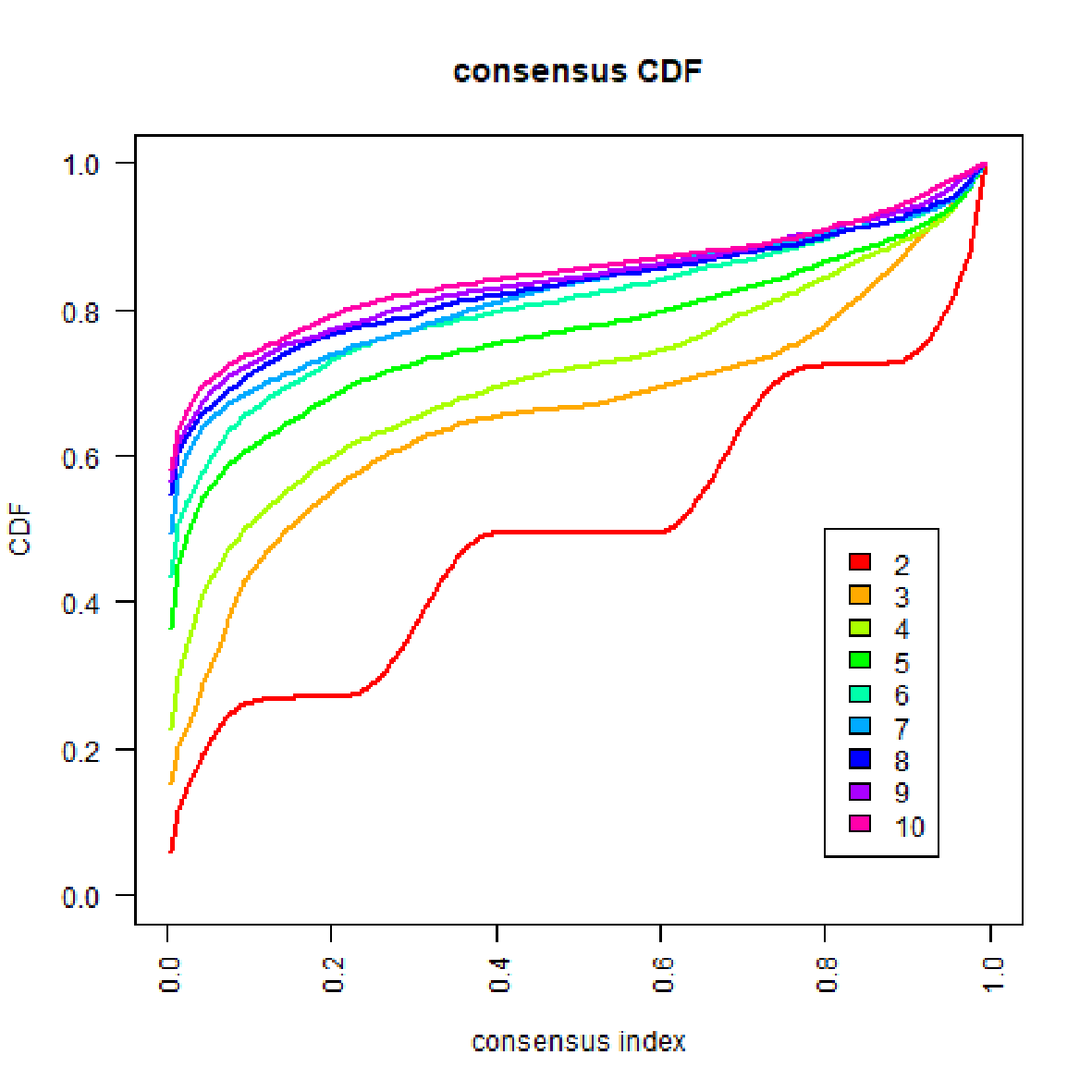

Supplement: Supplementary file 1 [file Image_1.PNG]

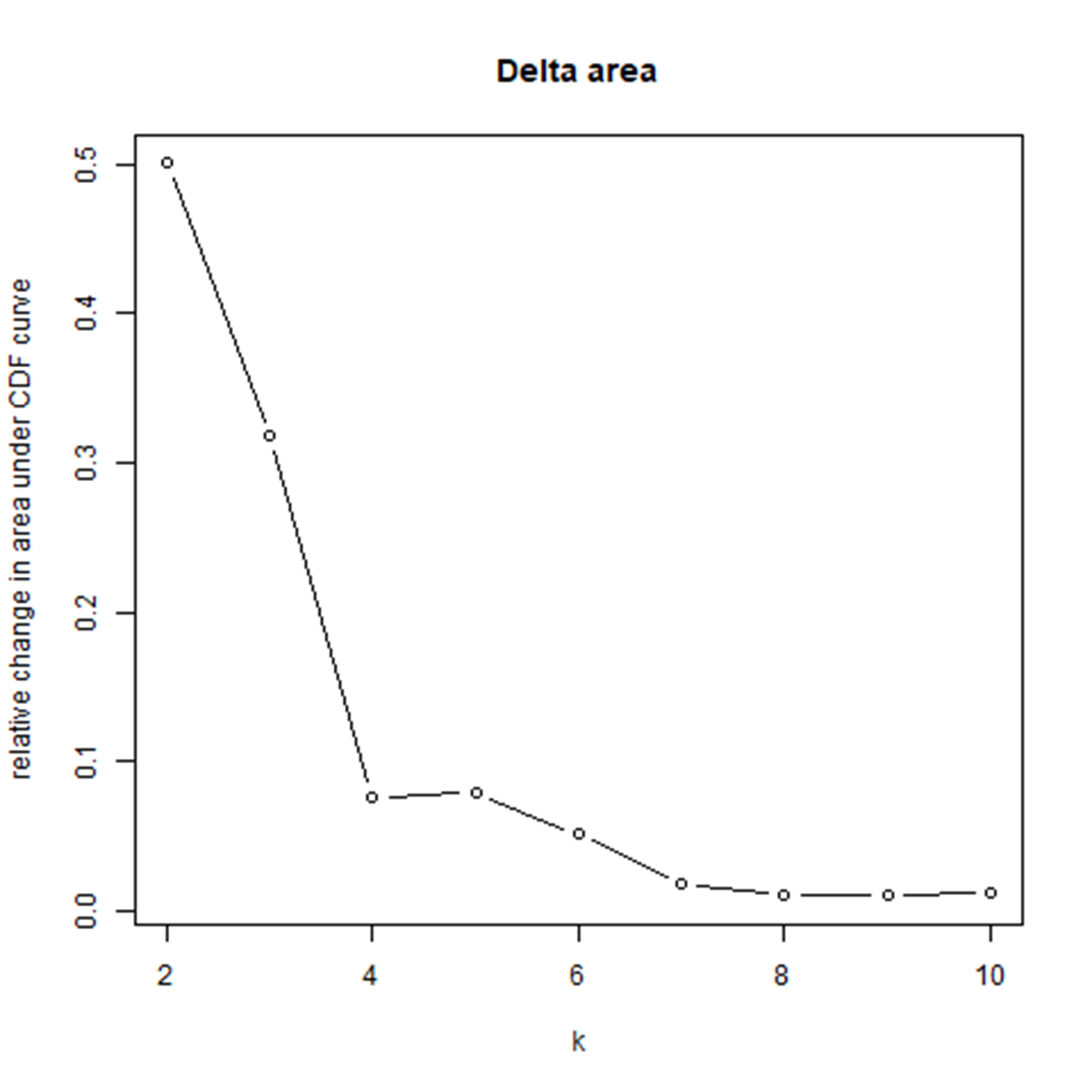

Supplement: Supplementary file 2 [file Image_2.PNG]

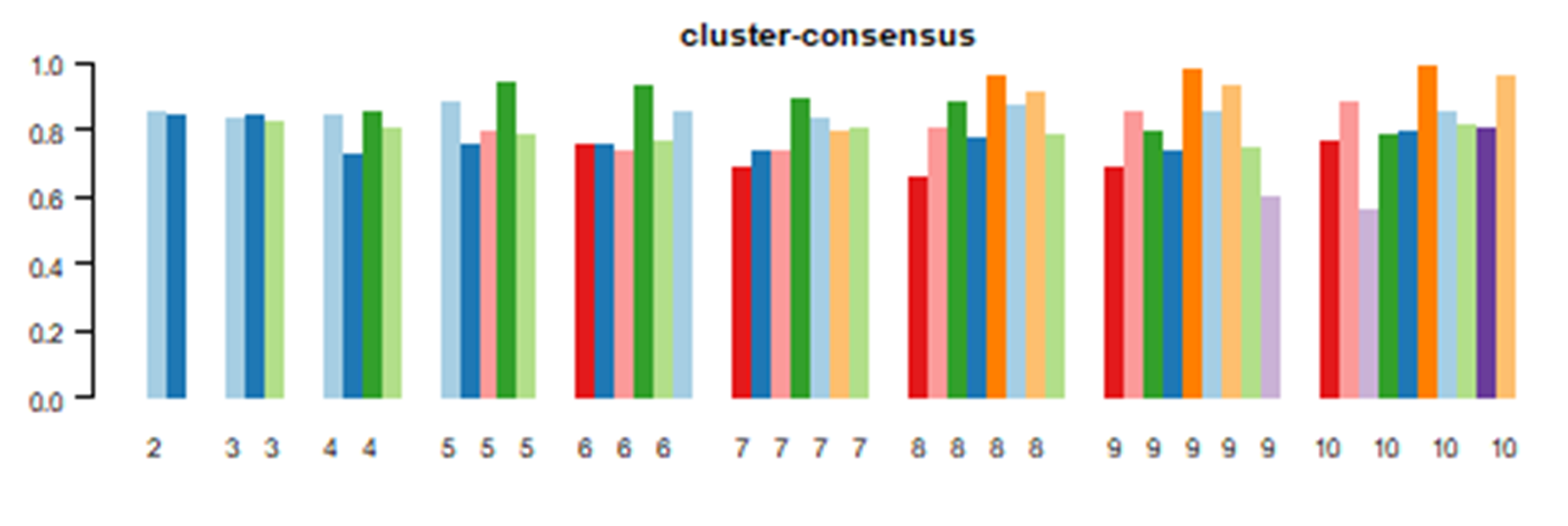

Supplement: Supplementary file 3 [file Image_3.PNG]

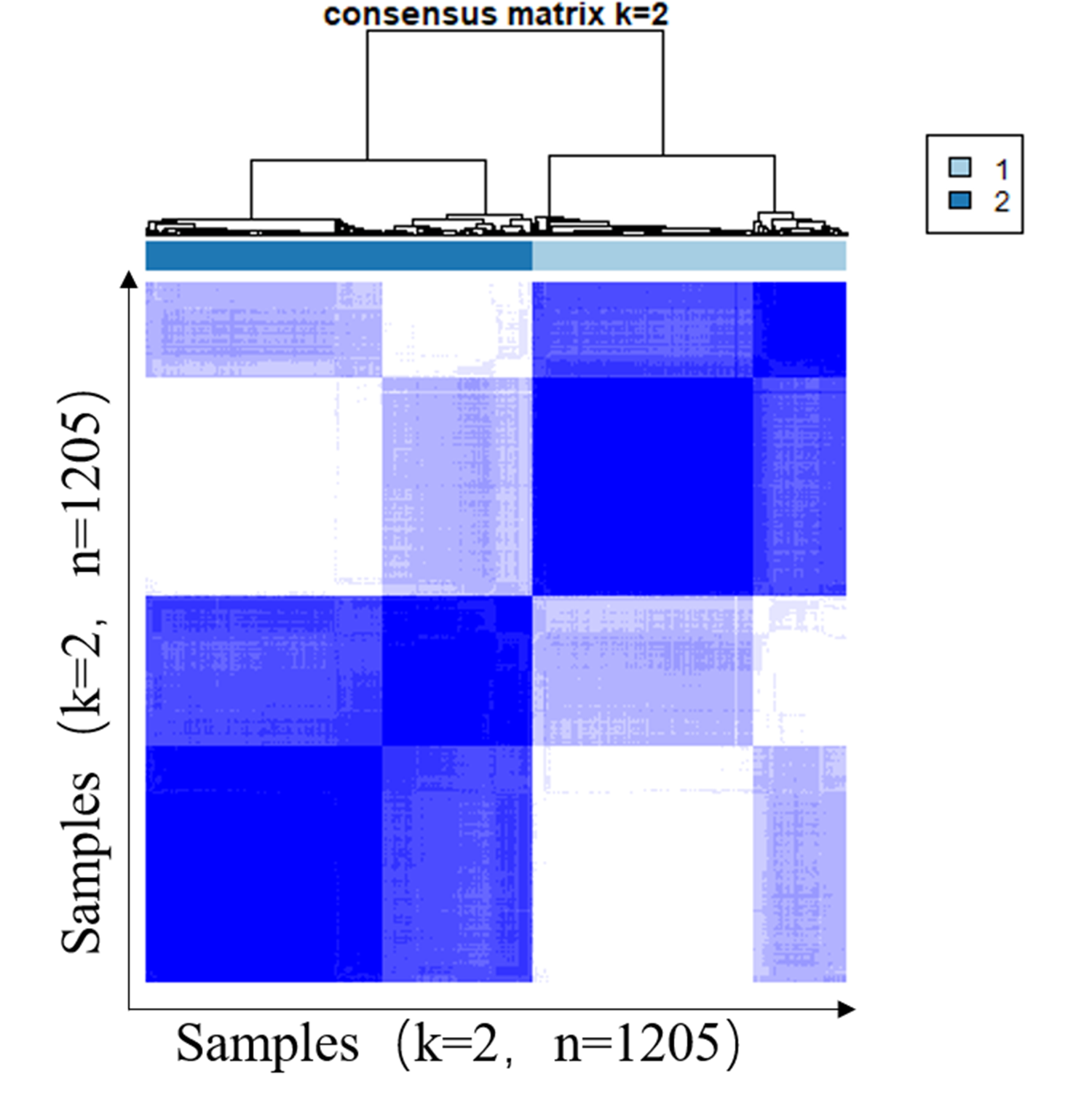

Supplement: Supplementary file 4 [file Image_4.PNG]

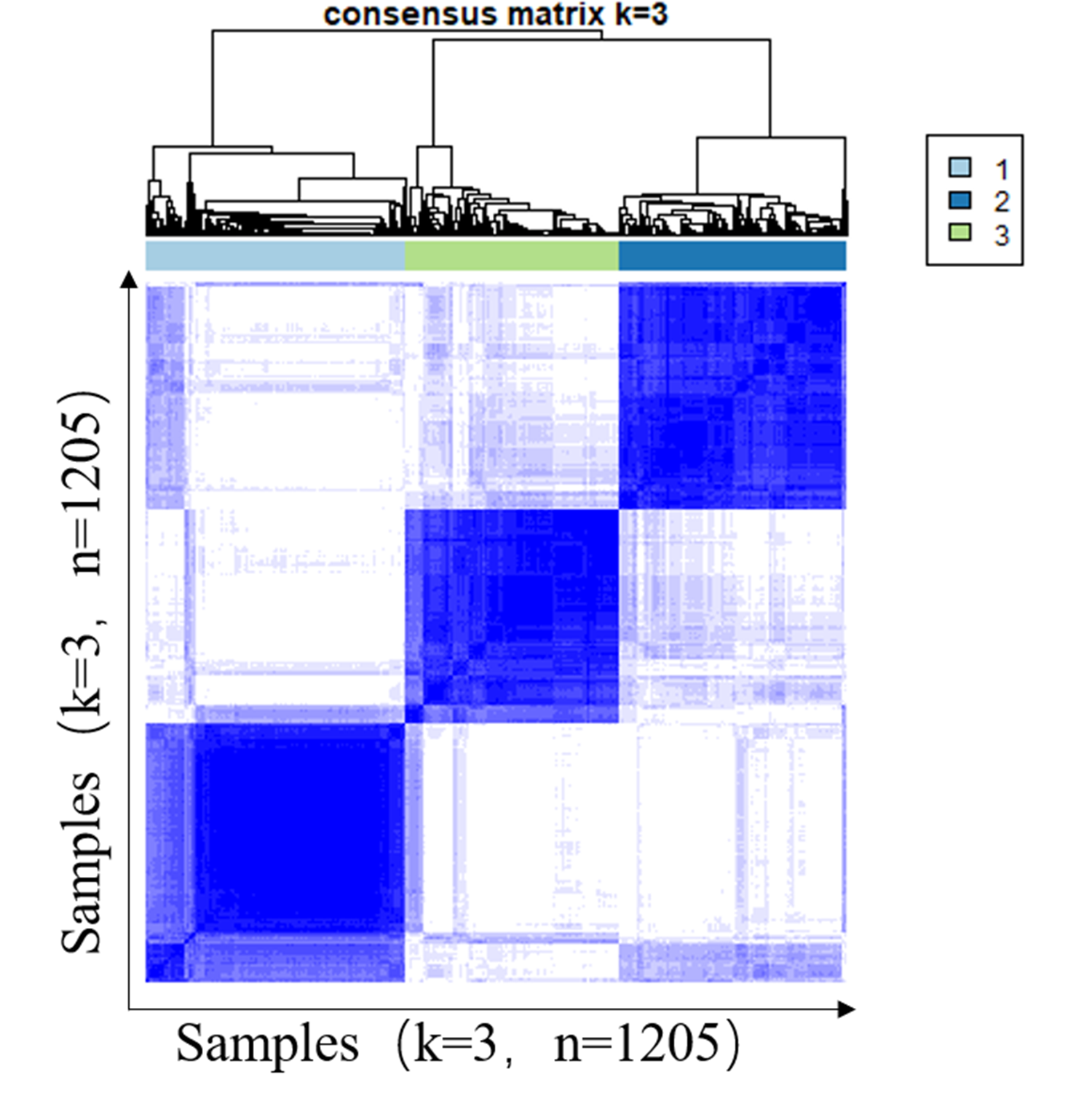

Supplement: Supplementary file 5 [file Image_5.PNG]

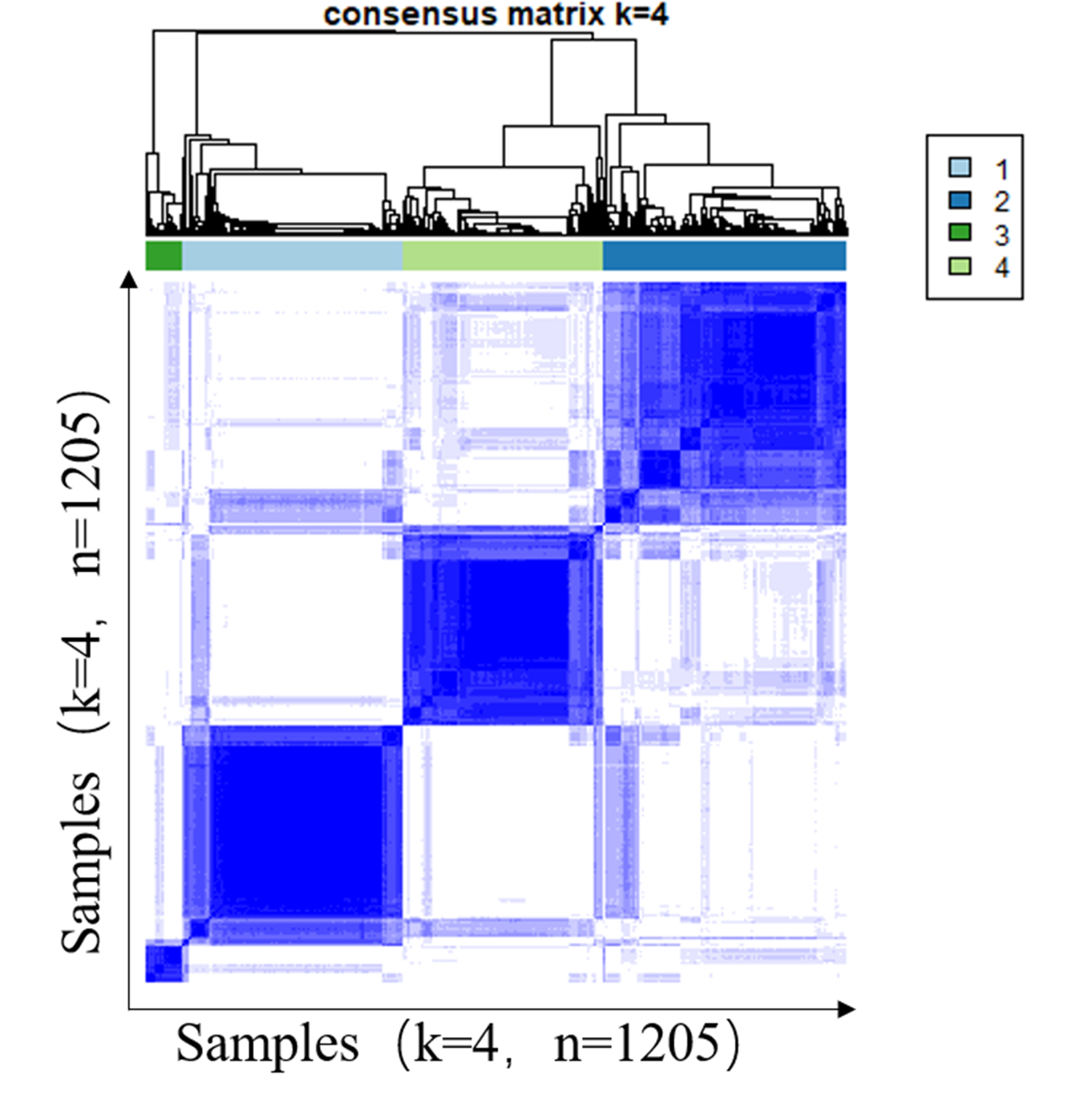

Supplement: Supplementary file 6 [file Image_6.PNG]

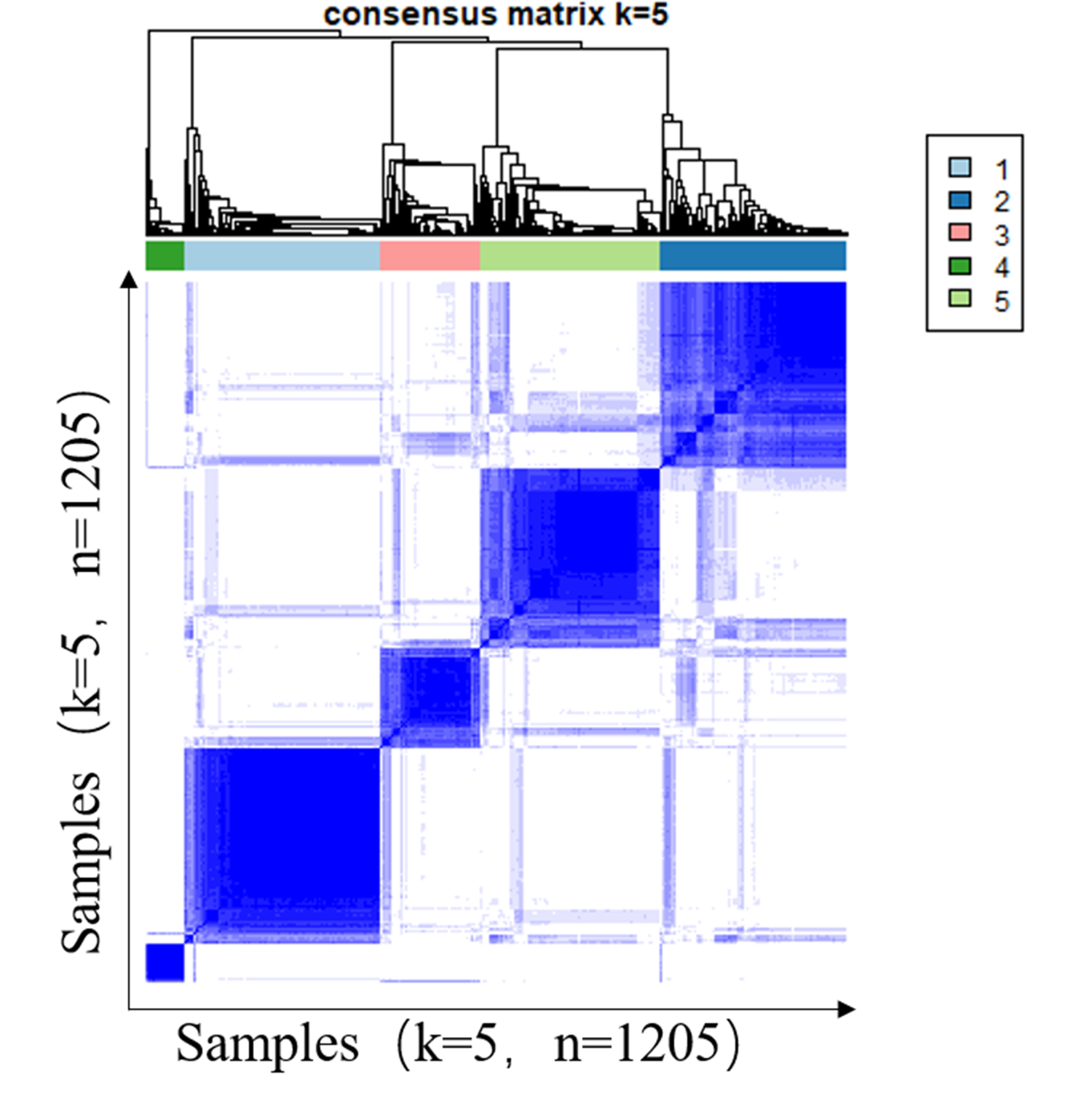

Supplement: Supplementary file 7 [file Image_7.PNG]

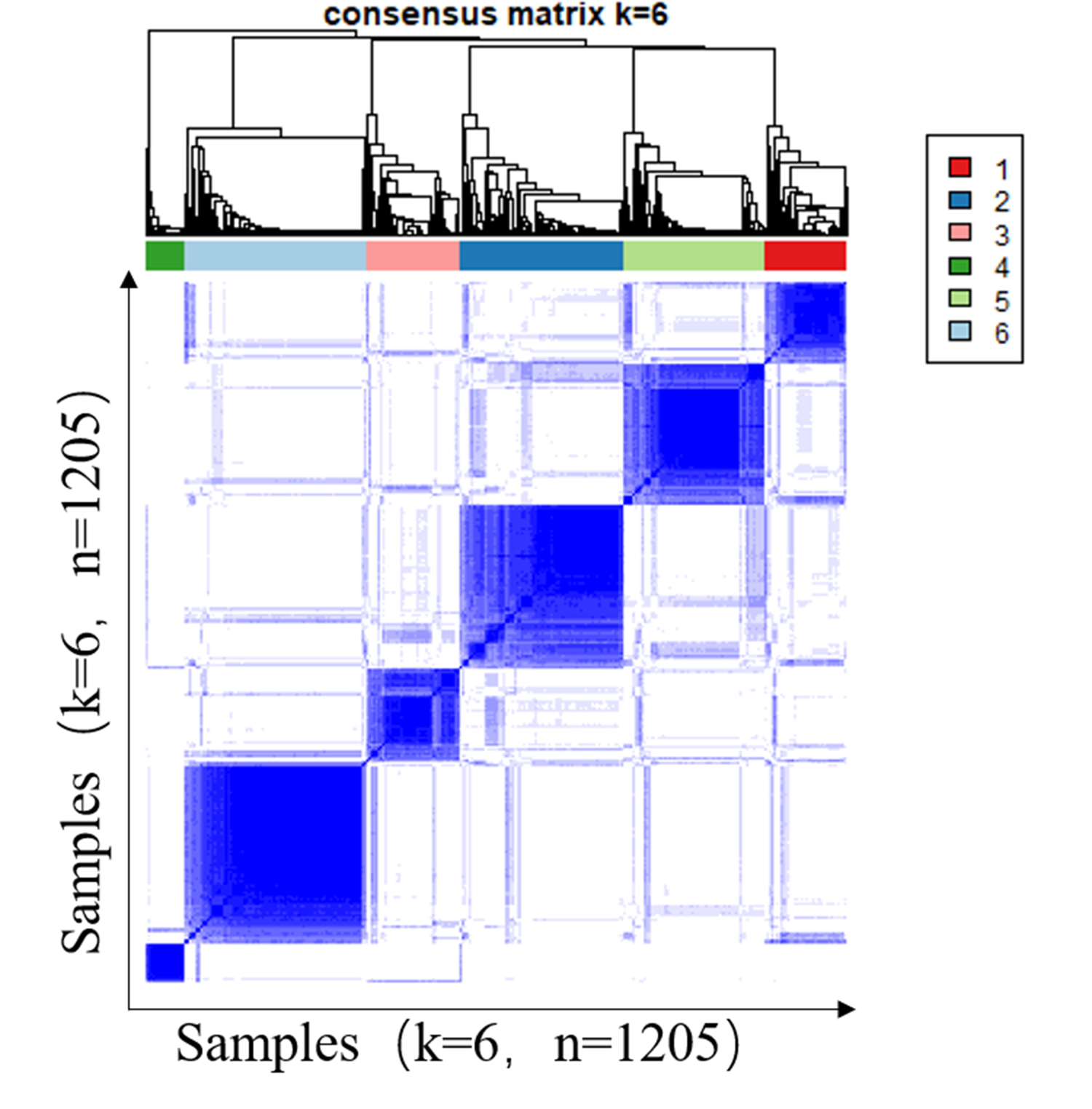

Supplement: Supplementary file 8 [file Image_8.PNG]

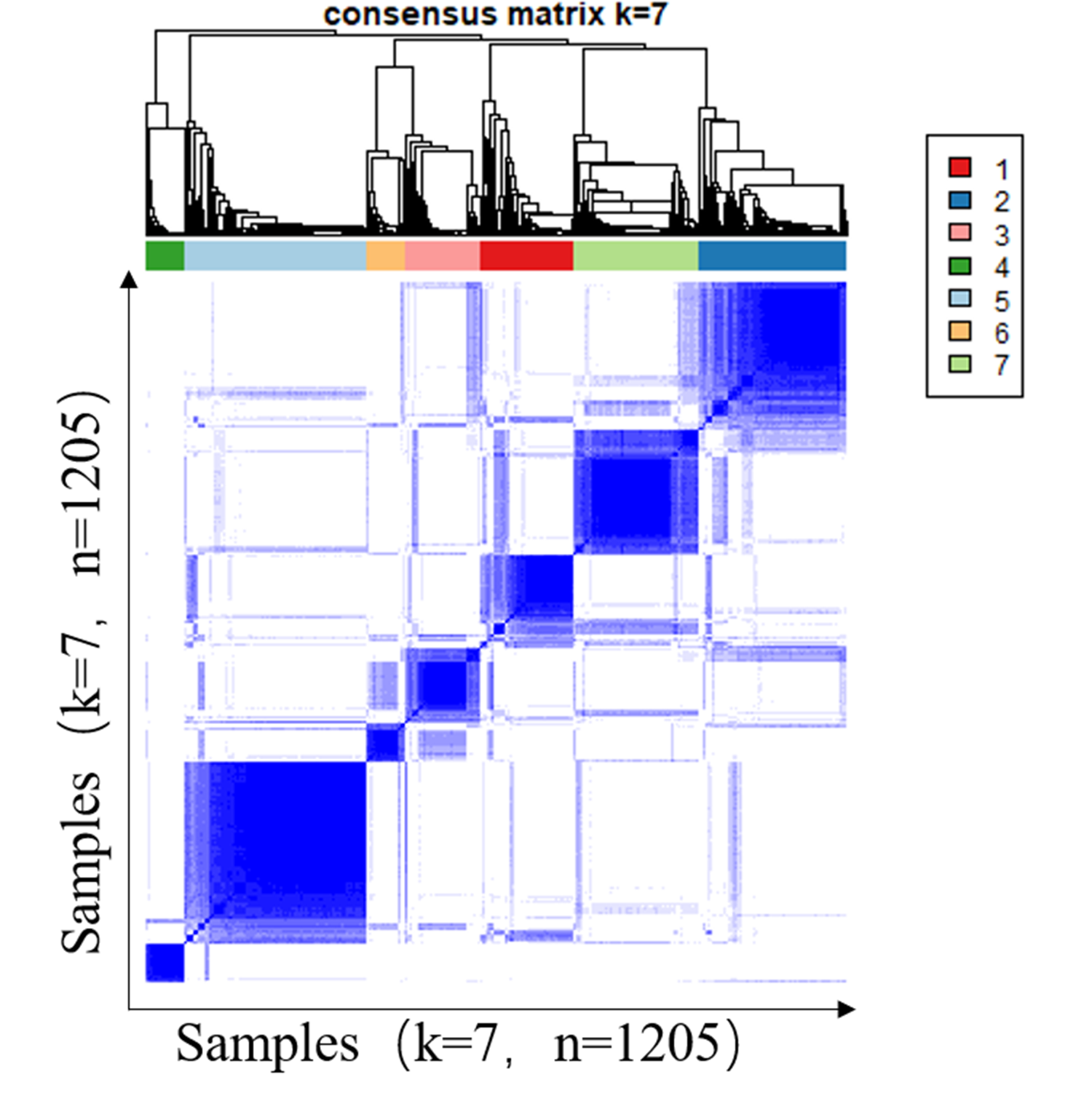

Supplement: Supplementary file 9 [file Image_9.PNG]

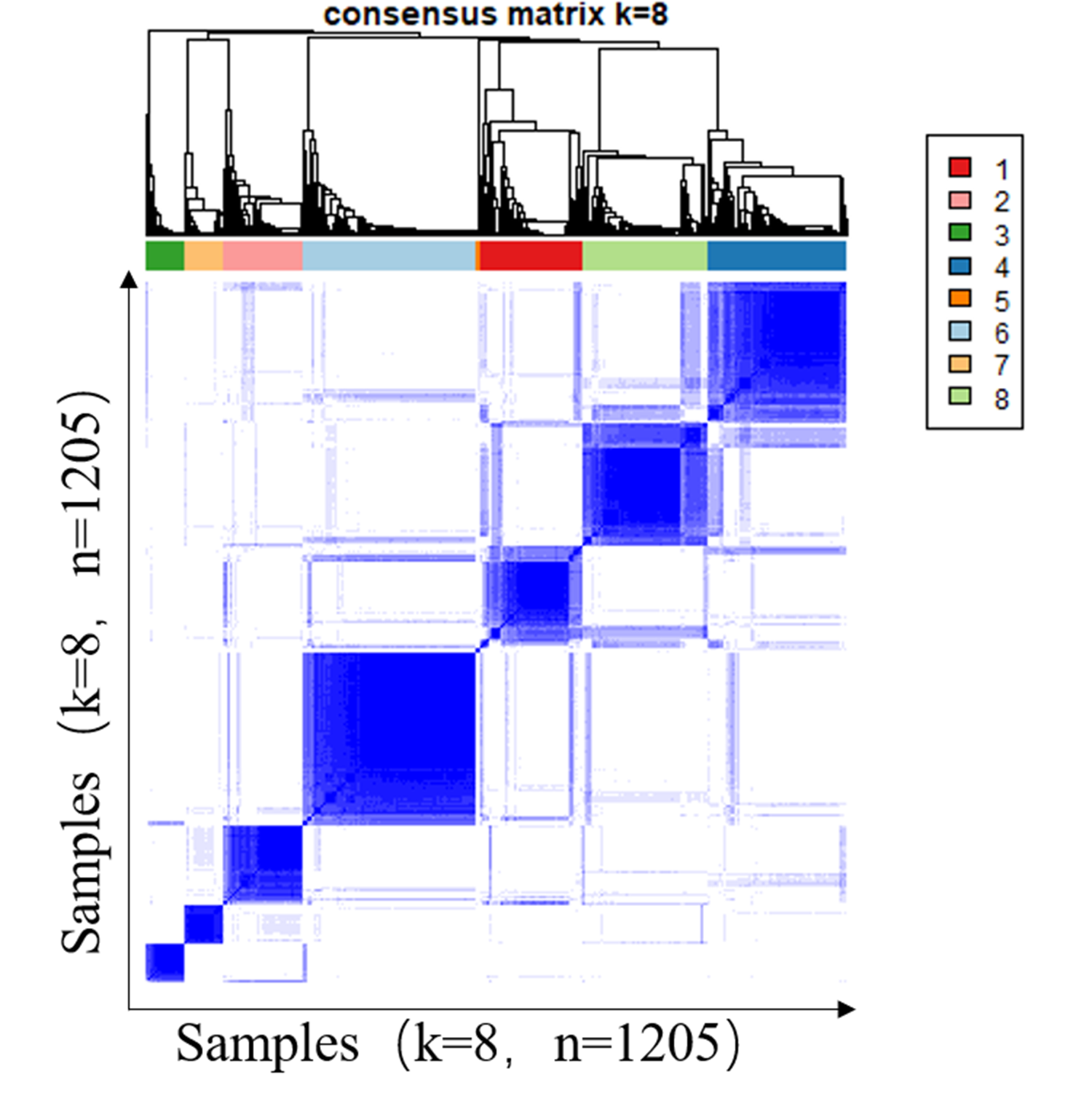

Supplement: Supplementary file 10 [file Image_10.PNG]

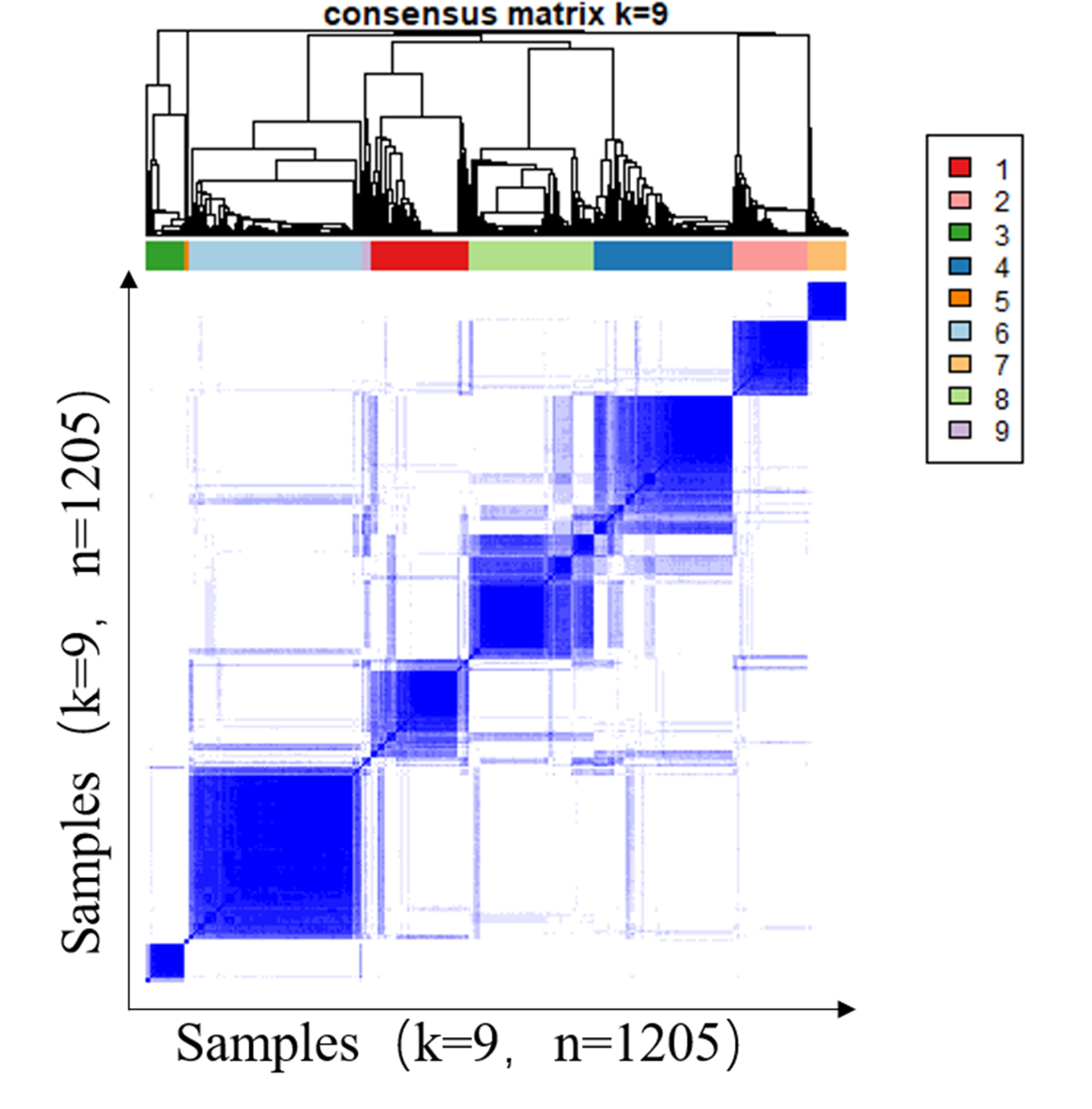

Supplement: Supplementary file 11 [file Image_11.PNG]

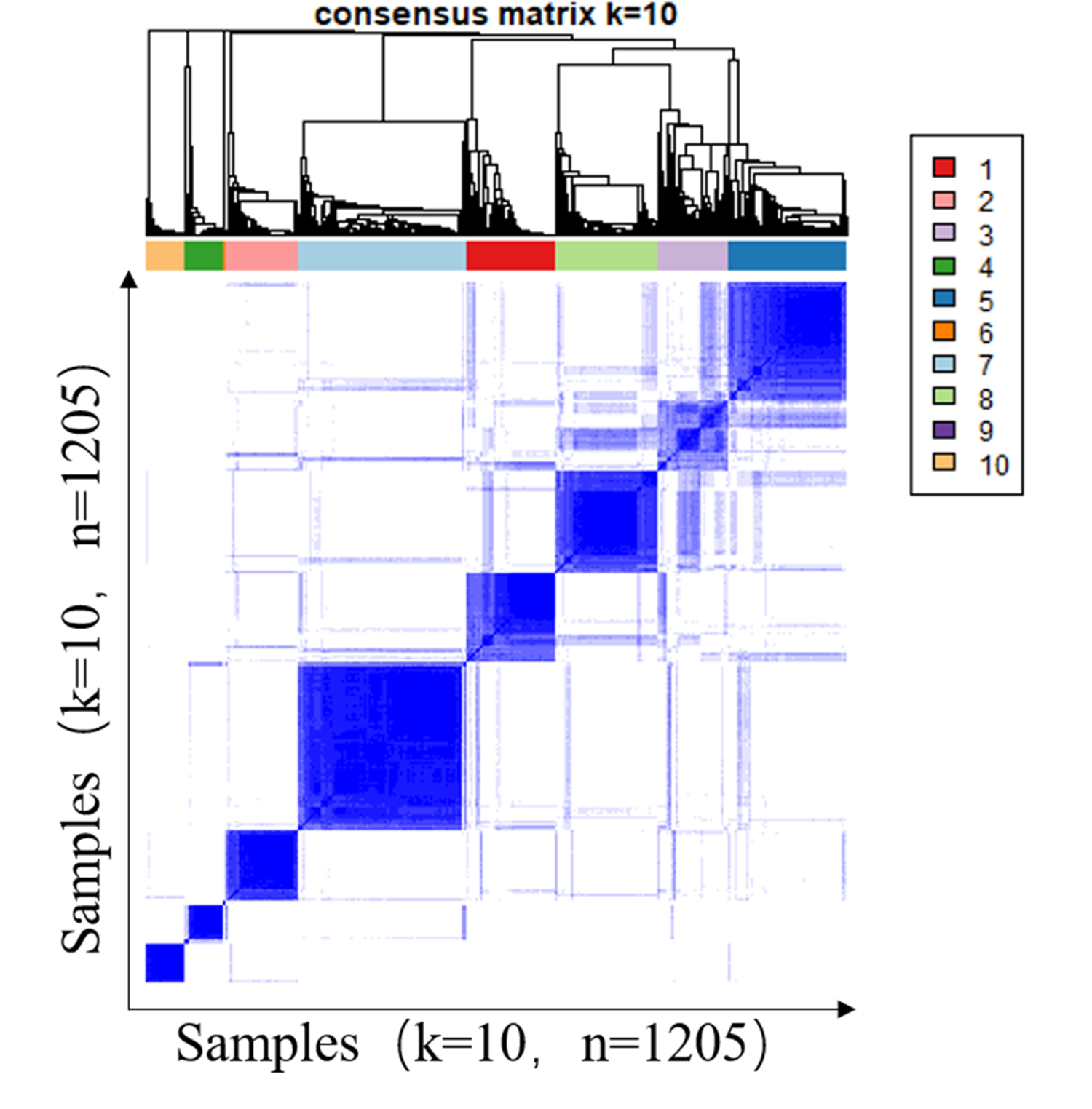

Supplement: Supplementary file 12 [file Image_12.PNG]
